# Third-Generation Solid Dispersion Through Lyophilization Enhanced Oral Bioavailability of Resveratrol

**DOI:** 10.1021/acsptsci.4c00029

**Published:** 2024-02-14

**Authors:** Hugo Almeida, Bárbara Ferreira, Carlos Fernandes-Lopes, Francisca Araújo, Maria João Bonifácio, Teófilo Vasconcelos, Bruno Sarmento

**Affiliations:** † ICBAS—Instituto de Ciências Biomédicas Abel Salazar, Universidade do Porto,Rua Jorge de Viterbo Ferreira, 228, 4050-313 Porto, Portugal; ‡ BIAL—Portela & Ca̲, S.A., Avenida da Siderurgia Nacional, 4745-457 Trofa, Portugal; § INEB—Instituto Nacional de Engenharia Biomédica, Universidade do Porto, Rua Alfredo Allen, 208, 4200-135 Porto, Portugal; ∥ i3S—Instituto de Investigação e Inovação em Saúde, Universidade do Porto, Rua Alfredo Allen, 208, 4200-135Porto, Portugal; ⊥ IUCS - CESPU, Rua Central de Gandra 1317, 4585-116 Gandra, Portugal

**Keywords:** resveratrol, bioavailability, solid dispersion, lyophilization, amorphous, permeability enhancer, metabolism inhibitor

## Abstract

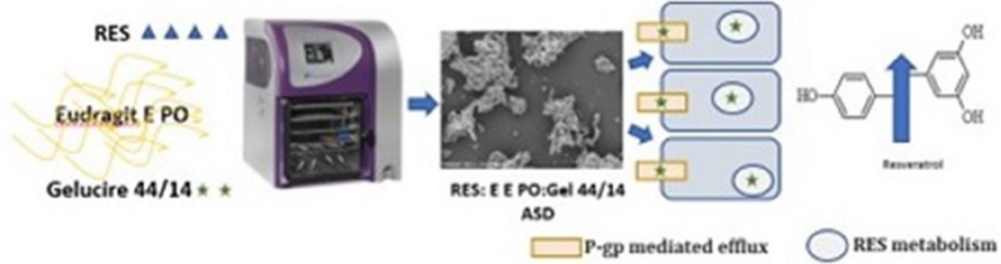

Resveratrol (RES) is a biopharmaceutical classification system (BCS) class II compound with low solubility and high permeability. Several strategies have been explored to overcome the low bioavailability of RES, making the formation of solid dispersions (SDs) one of the most promising. This study aimed at the development of a RES third-generation SD prepared by lyophilization as a strategy to improve RES solubility, dissolution, and oral bioavailability. Eudragit E PO was selected as the hydrophilic carrier in a 1:2 (RES:carrier) ratio, and Gelucire 44/14 as the surfactant, at 16% (w/w) of RES. Differential scanning calorimetry (DSC), scanning electron microscopy (SEM), Fourier-transform infrared spectroscopy (FTIR), polarized light microscopy (PLM), X-ray powder diffraction (XRPD), and particle size distribution (Morphologi 4 Malvern) were used for solid-state characterization and to confirm the conversion of RES to the amorphous state in the SD. Third-generation SD presented an 8-, 12-, and 8-fold increase of RES solubilized compared to pure RES at pH 1.2, 4.5, and 6.8, respectively, and a 10-fold increase compared to the physical mixture (PM), at pH 6.8, after 24 h. In the gastric environment, the dissolution rate of third-generation SD and PM was similar, and 2-fold higher than pure RES after 30 min, while at pH 6.8, third-generation SD presented approximately a 5-fold increase in comparison to pure RES and PM. Third-generation SD presented higher in vitro intestinal permeability compared to its PM and second-generation SD (without Gelucire 44/14). A 2.4 and 1.7-fold increase of RES permeated, respectively in Caco-2 and Caco-2/HT2-MTX models, was obtained with third-generation SD compared to PM, after 3 h. Third-generation SD allowed a 3-fold increase of RES bioavailability compared to second-generation SD, after oral administration of 200 mg/kg of RES to Wistar rats. Enhanced RES oral bioavailability was obtained not only by solubility and dissolution improvement, but also by the interference of Gelucire 44/14, with RES metabolism, and inhibition of P-gp-mediated efflux. The presence of excipients like Gelucire 44/14 in the SD allows for greater bioavailability of orally administered RES, making it easier to obtain some of the physiological benefits demonstrated by this molecule.

Resveratrol (3,5,4′-trihydroxystilbene), a nonflavonoid polyphenolic, is present in grapes, berries, and other plants. Resveratrol (RES) intake from dietary supplements and red wine has been shown to have several therapeutic properties.^1^ Most of the clinical trials involving RES have focused on cancer (prostate, breast, and colorectal),^2^ neurological disorders (Alzheimer’s disease and ischemic stroke),^3^ cardiovascular diseases (coronary artery disease, atherosclerosis, hypertension, and oxidative stress),^4,5^ diabetes (type 2 and impaired glucose tolerance), and nonalcoholic fatty liver disease.^6^

Despite these advantages, based on the Biopharmaceutical Classification System, RES is a class II compound with low solubility and high permeability.^7^ RES is extensively metabolized and rapidly eliminated and therefore it shows a poor bioavailability.^8^ After oral administration, RES is absorbed at a relatively high rate through the small intestine.^9^ The small and nonpolar character of RES may allow for its absorption across the membranes by passive diffusion, yet there is evidence that RES is mostly transported across the intestinal epithelium cell via ATP-dependent binding cassette (ABC) transporters.^10^

Strategies to increase bioavailability from oral delivery of RES are generally focused on increasing the rate of its absorption into the enterocytes and decreasing intracellular metabolism.^11^ Protecting RES from rapid metabolization in the gastrointestinal tract and liver is one general mechanism that can increase bioavailability.^1,12,13^

Solid dispersions (SDs) are one of the most successful strategies to improve drug release of poorly water-soluble drugs as described by Vasconcelos T, Sarmento B, Costa P.^14^ They enhance the oral absorption of poorly water-soluble drugs by attaining and sustaining a supersaturated concentration of drug in the gastrointestinal fluid. Formulation of poorly water-soluble compounds as SDs may lead to particle size reduction, improved wettability, reduced agglomeration, changeability in the physical state of the drug molecules, and possibly a dispersion in the molecular level, according to the physical state of the SD. Hot-melt-extrusion, spray-drying, and freeze-drying/lyophilization are common methods to prepare SDs.^14−17^

Freeze-drying or lyophilization is the process by which the solvent is removed from a frozen solution by sublimation. The freeze-drying process may be divided in three phases: freezing, primary drying (sublimation), and secondary drying (desorption).^14,18,19^ Most market products are lyophilized with aqueous solutions, however, some hydrophobic and insoluble drugs, such as RES, cannot be freeze-dried adequately with water-based formulations, so pure organic solvent or organic cosolvent + water formulations have also been investigated in recent years. *Tert*-butanol (TBA) a class 3 solvent with low toxicity, high vapor pressure, high melting point (±24 °C) and acceptable by FDA, is an excellent choice as a freeze-drying medium. The main advantages of using nonaqueous solvents are increased drug solubility, great acceleration of the sublimation rates, increased chemical stability of the predried bulk solution, increased chemical stability of the dried product, and facilitated manufacture of the bulk solution by increasing drug wettability and solubility in solution.^20^

Third-generation SDs contain a surfactant matrix or a mixture of amorphous polymers and surfactants as carriers. With this approach, the aim is to reach the maximum bioavailability for poorly water-soluble drugs and stabilize the SD, preventing drug recrystallization.^14,21−23^

The use of surfactants such as inulin, Compritol 888 ATO, Gelucire 44/14, and Poloxamer 407, demonstrated efficacy in producing high polymorph purity and in the improvement of oral bioavailability. The inclusion of surfactants in the formulation containing a polymeric matrix can help prevent precipitation or protect a fine crystalline precipitate from agglomeration into much larger hydrophobic particles.^14,21,23−27^

Based on the above, the development of a RES third-generation SD prepared by lyophilization was the strategy pursued to improve its oral bioavailability. Initially, excipients selection and optimization were performed by batch lyophilization (in vials), using different cosolvent systems based on the solubility constraints caused by formulation composition throughout the drug delivery system development process. After final formulation selection, SDs were manufactured by bulk lyophilization and completely characterized for solid state, solubility, dissolution, in vitro intestinal permeability, and in vivo pharmacokinetics.

## 1 Results and Discussion

### 1.1 Resveratrol Solid Dispersion Development

#### 1.1.1 Selection and Optimization of the Hydrophilic Carrier Content

Aiming to develop an RES drug delivery system with improved solubility and consequently enhanced oral bioavailability, by the solvent evaporation method (lyophilization/freeze-drying), several hydrophilic polymers were initially screened and characterized with the purpose of dispersing the RES at a molecular level and consequently induce its solubility improvement. Nonionic polymers: polyethylene glycol (PEG 10000); polyvinylpyrrolidone (Povidone K30); copovidone (Plasdone S-630); polyvinyl caprolactam-polyvinyl acetate-polyethylene glycol graft copolymer (Soluplus); hydroxypropyl methyl cellulose (HPMC; MW: 1261.4), and hydroxypropyl cellulose low viscosity (HPC SL; MW: 806.9). Cationic polymer: cationic methacrylate copolymer (Eudragit E100). Anionic polymer: hypromellose acetate succinate (HPMC AS-MG).

##### 1.1.1.1 Hydrophilic Carrier Selection

*Resveratrol:Polymer (1:1) in TBA/Water (70:30)*: When Kollidon k30 previously dissolved in water was added to RES dissolved in TBA, precipitation occurred. For this reason, Kollidon k30 was abandoned as a possible choice for hydrophilic polymer selection. After lyophilization, intact wafers with good appearance were obtained with HPMC alone. Wafers collapsed during freeze-drying with all other polymers tested. Probably, the solid content was too low (2% w/v) in those formulations, to produce a wafer with good mechanical strength. It was decided to add mannitol at 4% (w/v) as a bulking agent. Mannitol as a commonly used lyoprotectant prevents structural collapse during freeze-drying and enhances mechanical properties.

*Resveratrol:Polymer:Mannitol (1:1:8) in TBA/Water (50:50)*: After lyophilization, intact wafers with good appearance were obtained for all polymers tested. Solubility for RES, physical mixtures (PMs), and successful lyophilized formulations (LFs) from phase a) and b), at pH 1.2 and 6.8 was assessed (Supporting Information). RES was very low soluble in both aqueous solvents of 53.4 μg/mL (pH 1.2) and 52.0 μg/mL (pH 6.8) respectively. Eudragit E100 presented the highest increase in solubility for both PM and LF at pH 1.2. At pH 6.8, solubility for the PM was similar to RES alone, while for LF solubility was 264.5 μg/mL. Eudragit E100, being a cationic polymer, precipitates above pH 5.5, explaining the low solubility of RES obtained in the PM at pH 6.8. Additionally, it was difficult to micronize and dissolve. Another cationic methacrylate copolymer (Eudragit E PO) in the form of a dry powder, was further used to overcome the difficulties observed with Eudragit E100.

##### 1.1.1.2 Hydrophilic Carrier Content Optimization

**Figure 1:**
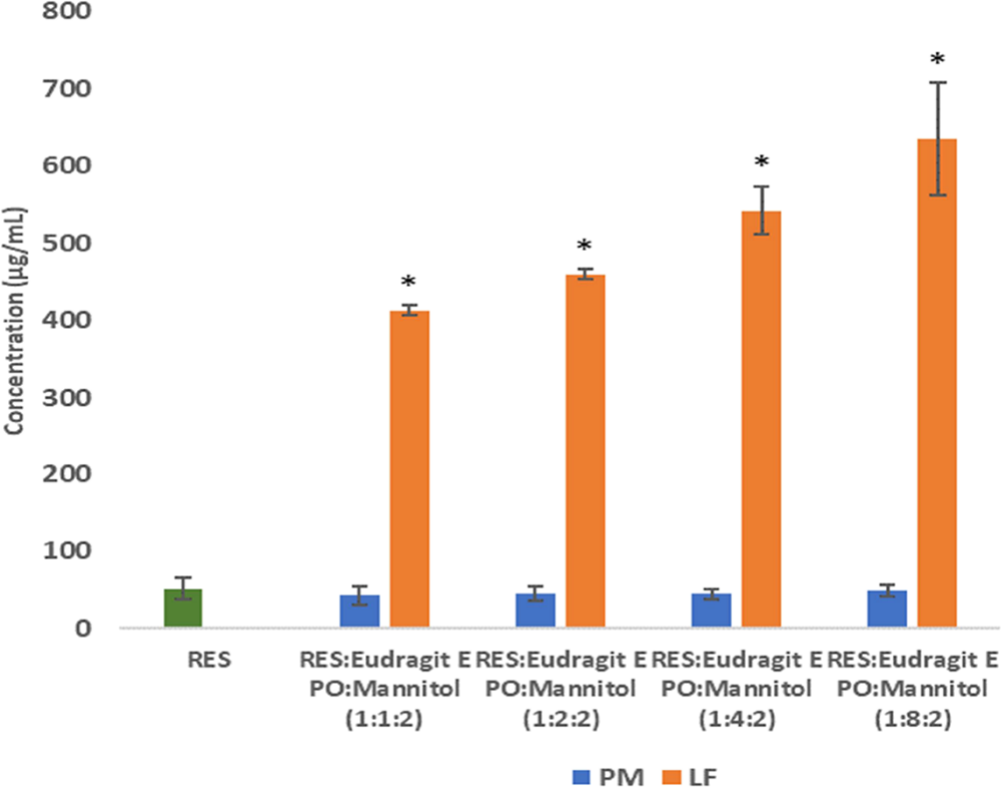
Solubility after 24 h under magnetic stirring at room temperature in pH 6.8 (Eudragit E PO used at different ratios in formulation). (mean ± SD, *n* = 3), *p < 0.05 comparing with pure RES and PMs.

Eudragit E PO was tested at different ratios with RES aiming to decrease mannitol content without compromising the wafer's appearance and mechanical strength. After lyophilization, intact wafers with good appearance were obtained for all formulations. Solubility for PMs and LFs at pH 6.8 was assessed as presented in Figure 1.

Eudragit E PO is a nonhygroscopic hydrophilic cationic polymer consisting of methyl methacrylate, N–N-dimethylaminoethyl methacrylate, and butyl methacrylate monomers (1:2:1) and possesses tertiary amines that ionize at the acidic pH to make the polymer highly soluble in fluids when pH is below 5.^28^ A huge increase in RES solubility was observed for all LFs with values above 400 μg/mL. In all PMs the solubility results are similar to the solubility of RES alone, also confirming the results obtained with Eudragit E100 at pH 6.8.

Based on the results obtained, RES:Eudragit E PO:Mannitol (1:1:2) was the selected formulation at this moment. Wafers with good appearance and mechanical strength were obtained with this formulation. Although an increase in the polymer portion yields higher solubility results, the mass of the SD in a future final solid dosage form may be very high. The aim for the next phase was the selection of a surfactant to stabilize the SD and improve solubility allowing an enhanced bioavailability of RES.

#### 1.1.2 Selection and Optimization of the Surfactant Content

##### 1.1.2.1 Surfactant Selection

Compitrol 888 ATO, Docusate sodium, Gelucire 44/14, Polaxamer 407, Tween 80, Labrasol, Cetrimide, sodium dodecyl sulfate (SDS), and Kolliphor RH 40 were the surfactants tested at 4% (w/w) of RES. Solubility in pH 1.2 aqueous media for LFs was assessed at T0 and after 1 month at 40 °C/75% RH (Supporting Information). At T0 at least a 2-fold increase in solubility was observed for all LFs with surfactant compared to the formulation without surfactant (151.3 μg/mL). The lowest increase in RES solubility was observed with Tween 80 (352.4 μg/mL), while higher average values were obtained with cetrimide (498.2 μg/mL), docusate sodium (401.8 μg/mL) and Gelucire 44/14 (401.7 μg/mL).

After 1 month at 40 °C/75% RH, a decrease in RES solubility was observed for all LFs, with the greatest average reduction observed with docusate sodium (401.8 μg/mL to 172.4 μg/mL). The formulations with cetrimide and Gelucire 44/14 were among those that showed the smallest decrease in solubility and the only ones that maintained average values above 300 μg/mL, (Gelucire 44/14–300.1 μg/mL; cetrimide −379.1 μg/mL).

Although cetrimide presented slightly higher solubility results compared to Gelucire 44/14, this was the surfactant selected, also considering its effect on presystemic drug metabolism inhibition and interference in P-gp mediated efflux, which contribute to an improved RES bioavailability.^26,29,30^ Gelucire 44/14 is also a nonionic surfactant with a less irritant effect than a cationic surfactant such as cetrimide.^31^

Gelucire 44/14 is a nonionic water-dispersible surfactant obtained by an alcoholysis reaction between coconut oil and polyethylene glycol-32 (PEG-32) under controlled conditions. It consists of glycerides and PEG esters of fatty acids of varying chain lengths.^32^

##### 1.1.2.2 Surfactant Content Optimization

**Figure 2:**
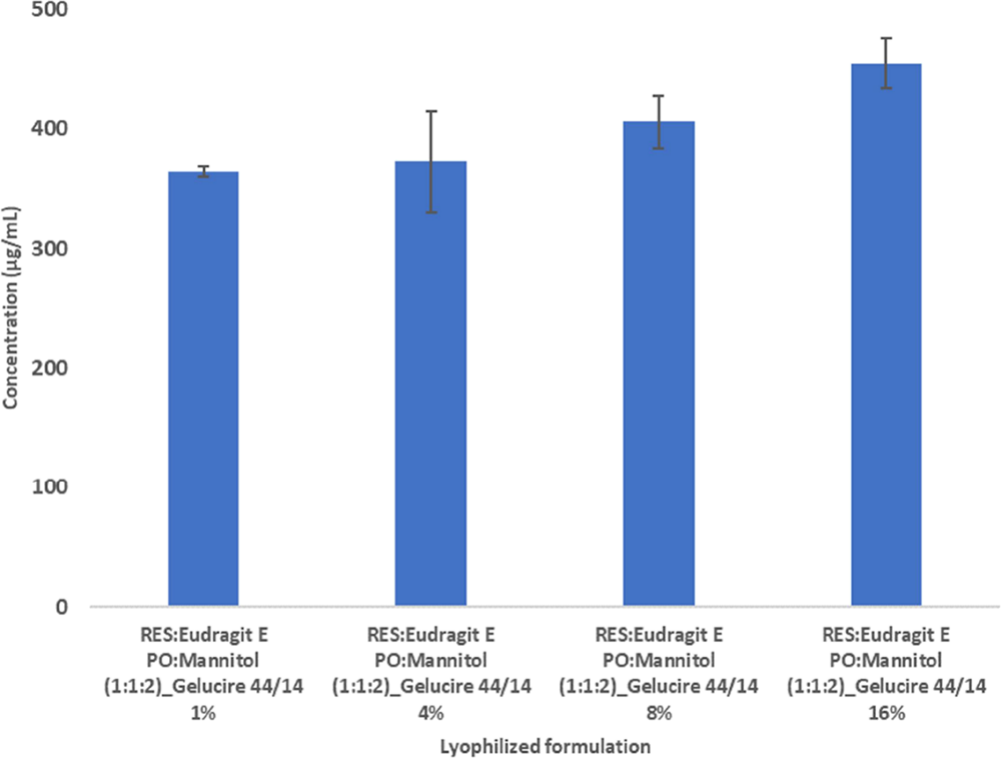
Solubility after 24 h under magnetic stirring at room temperature in pH 1.2 (Gelucire 44/14 at 1, 4, 8, and 16% w/w of RES). (mean ± SD, *n* = 3).

Lyophilized formulations with Gelucire 44/14 at different concentrations, 1, 8, and 16% (w/w) of RES were produced and compared to the previous one produced with 4% (w/w) to assess the impact of surfactant content in RES solubility at pH 1.2 as presented in Figure 2.

RES solubility slightly increased with the percentage of surfactant. Gelucire 44/14 at 16% (w/w) of RES was selected for the formulation, also considering that a greater amount can potentiate its effect on presystemic drug metabolism inhibition and interference in P-gp mediated efflux, which contributes to an improved RES bioavailability.^26,29,30^

#### 1.1.3 Bulking Agent

At the beginning of formulation development wafers collapsed during freeze-drying and mannitol was selected as a bulking agent, since is a commonly used excipient as a lyoprotectant in freeze-drying. Bulking agents, such as mannitol and lactose, are utilized in lyophilized formulations to provide structure to the lyophilized cake, preventing collapse. Nevertheless, it is important to understand that bulking agents can be dangerous for lyophilized products. For example, when using mannitol, it is essential to ensure that it is fully crystallized. If mannitol crystallizes postlyophilization, it can release the water associated with it back into the cake, potentially accelerating the destabilization of the product.^33,34^

**Figure 3:**
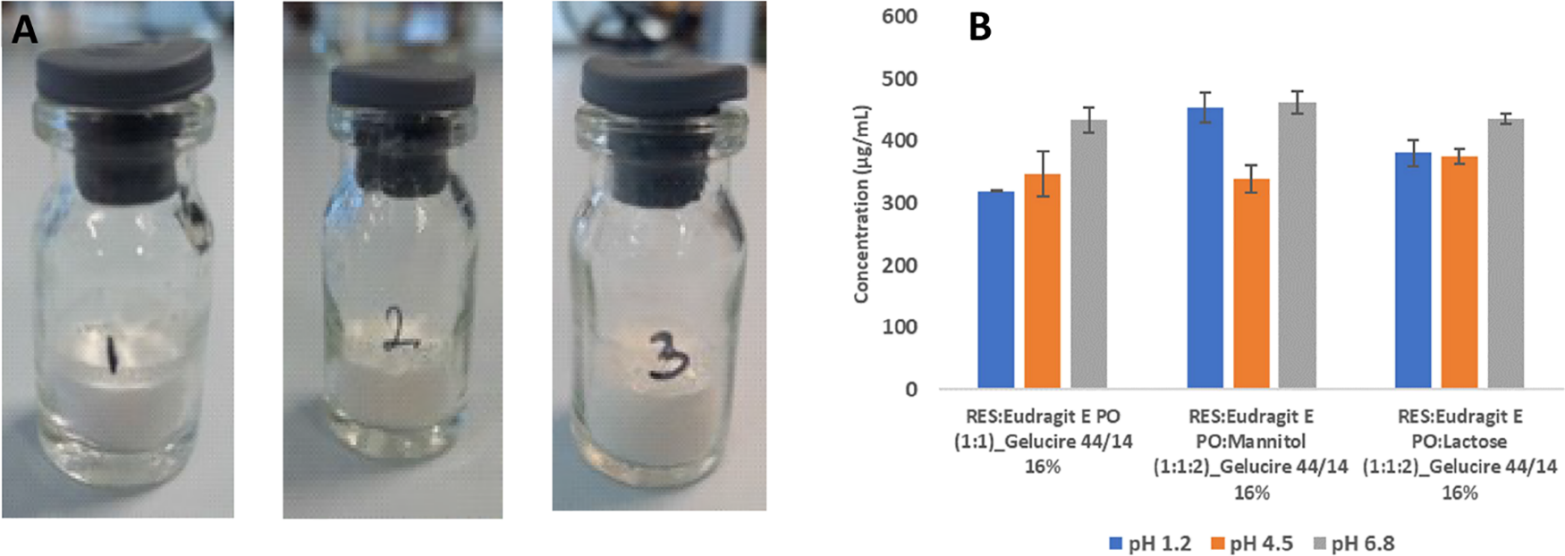
RES:Eudragit E PO:Mannitol (1:1:2)_Gelucire 44/14 16% (1); RES:Eudragit E PO:Lactose (1:1:2)_Gelucire 44/14 16% (2); RES:Eudragit E PO (1:1)_Gelucire 44/14 16% (3) lyophilized wafers (A). Solubility after 24 h under magnetic stirring at room temperature in pH 1.2, pH 4.5 and pH 6.8. (mean ± SD, *n* = 3) (B).

The presence of a bulking agent will also increase the mass of the SD formulation with an impact on the size of the final oral solid dosage form. Based on the above, it was assessed if the formulation selected (now, including the surfactant) presents sufficient mechanical strength and good wafer appearance when removing mannitol as a bulking agent. Formulation with lactose as an alternative to mannitol was also tested.^35^ After lyophilization intact wafers with good appearance were obtained for all formulations tested as presented in Figure 3A.

Solubility was similar in all aqueous media for formulations without bulking agents and lactose or mannitol as bulking agents. Intact wafers with good appearance and adequate mechanical strength were obtained in the formulation without a bulking agent, suggesting that there is no need for lyoprotectant. Considering the previous, Resveratrol:Eudragit E PO (1:2)_Gelucire 44/14 16% was the formulation selected to be tested and produced by the bulk method. The removal of the lyoprotectant allowed an increase in the percentage of Eudragit E PO in the formulation without increasing the final mass of the LF, which could be too high to develop an oral solid dosage form with favorable patient compliance. The bulk lyophilization method was performed in aluminum trays allowing the production of larger quantities of SD for inclusion in oral solid dosage forms.

### 1.2 Solid Dispersions Characterization

#### 1.2.1 Differential Scanning Calorimetry (DSC), X-Ray Powder Diffraction (XRPD) and Fourier Transform Infrared Spectroscopy (FTIR)

**Figure 4:**
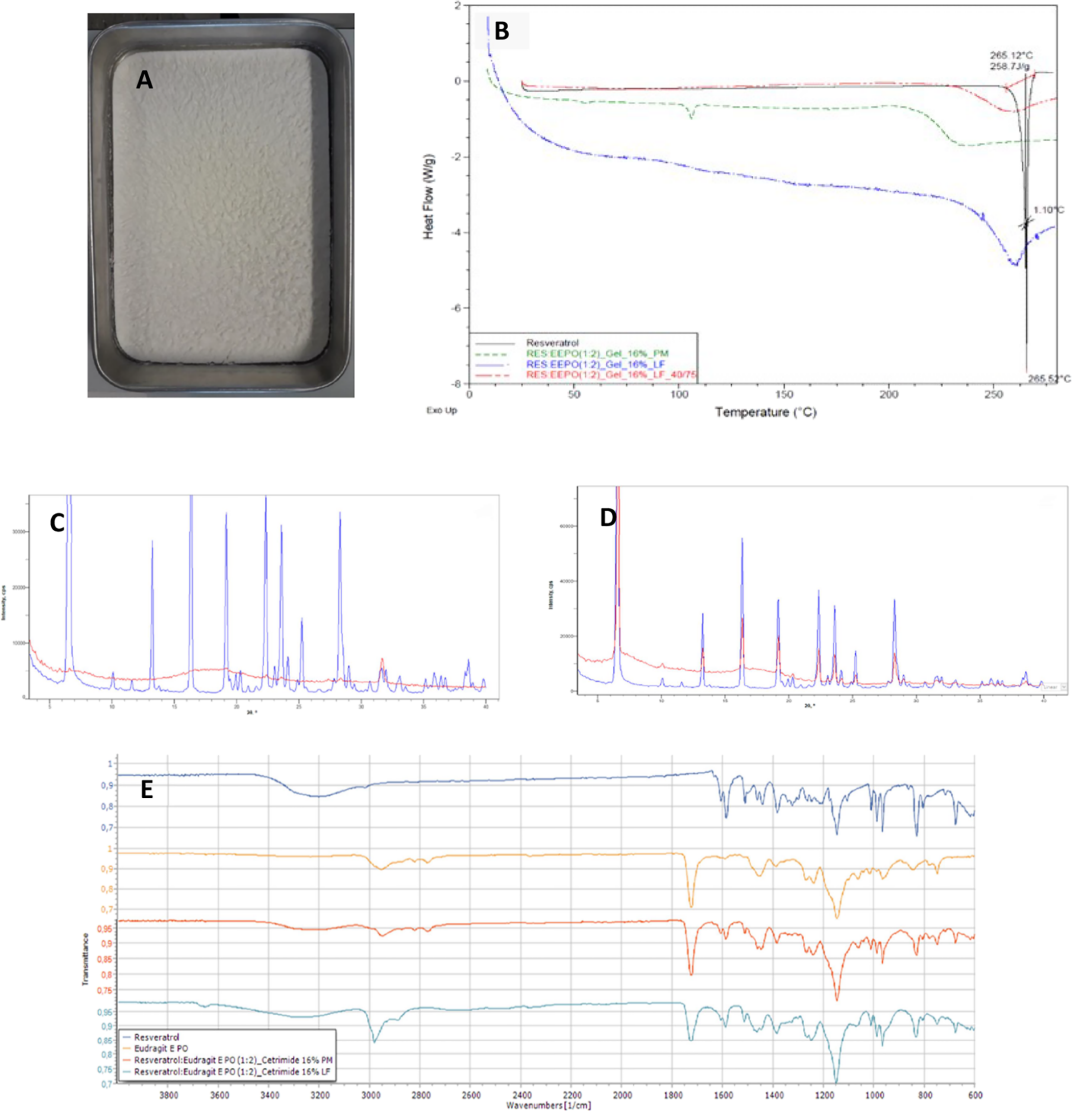
RES:Eudragit E PO (1:2)_Gelucire 44/14 16% SD powder (A). RES and RES:Eudragit E PO (1:2) Gelucire 44/14 16% thermograms (PM and LF at T0 and T1 month at 40 °C/75% RH) (B). XRPD patterns of RES (blue) and RES:Eudragit E PO (1:2)_Gelucire 44/14 16% SD (red) (C). XRPD patterns of RES (blue) and RES:Eudragit E PO (1:2)_Gelucire 44/14 16% PM (red) (D). FTIR spectra for RES, Eudragit E PO and RES:Eudragit E PO (1:2)_Gelucire 44/14 16% (PM and SD/LF) (E).

RES SD with Eudragit E PO and Gelucire 44/14 (third-generation SD; Figure 4A) and with Eudragit E PO alone (second-generation SD) were prepared by bulk lyophilization. This method allowed us to obtain a uniform powder layer with good appearance, not cracked, collapsed, or melted.

RES, RES:Eudragit E PO (1:2)_Gelucire 44/14 16% physical mixture (PM) at T0, and RES:Eudragit E PO (1:2)_Gelucire 44/14 16% solid dispersion (SD)/lyophilized formulation (LF) at T0 and after 1 month at 40 °C/75% RH were assessed by DSC (Figure 4B). The RES thermogram shows a single and sharp endothermic peak at 265.52 °C, which corresponds to its melting point with an enthalpy value of 258.7 J/g. In LF/third-generation SD thermogram at T0, a small endothermic event can be observed around the melting temperature of RES, which did not increase after 1 month at 40 °C/75% RH. The pronounced reduction in the endothermic event compared to RES alone, and the fact that it did not increase during the stress study, can indicate amorphization and improved solubility for RES in the LF, which was confirmed in the solubility assessment.

RES alone showed characteristic sharp diffraction peaks at 2θ, which highlights its crystallinity, (Figure 4C,D, in blue). In RES:Eudragit E PO (1:2)_Gelucire 44/14 16% PM, (Figure 4D, in red), there was no conversion of RES to the amorphous state, indicated by the presence of characteristic RES peaks. However, these peaks were not detected for RES:Eudragit E PO (1:2)_Gelucire 44/14 16% SD, (Figure 4C, in red), suggesting conversion of RES from the crystalline to the amorphous state, despite the presence of a small peak at around 31–32°, which do not correspond to any peak in the RES alone nor in PM XRPD patterns. Its origin can be possible explained by some sodium remnants from the solvent in the formulation that was not completely removed.

RES showed a broad peak at 3203 cm^–1^, that was assigned to the phenolic hydroxyl group stretch. Three sharp peaks at 1584, 1510, and 1461 cm^–1^, correspond to the aromatic skeleton vibration. RES exists in *cis*- and *trans*-form, having *trans*-RES higher biological activity. The peak at 964.5 cm^–1^ was attributed to the out-plane vibration of the double-bond carbon of *trans*-RES (Figure 4E). Eudragit E PO presents a peak at 1723 cm^–1^ that was attributed to the carboxyl group (Figure 4E). In both the PM and SD spectra, the broad peak attributed to the phenolic hydroxyl group of RES decreased intensity or slightly shifted (Figure 4E). This may be attributed to the dilution effect of the polymer in the PM and/or additionally to the establishment of hydrogen bonds with the carboxyl group of the polymer. Moreover, the Eudragit E PO peaks at 2820 and 2769 cm^–1^ corresponding to the nonionized demethylamino groups nearly disappeared in the SD/LF, suggesting the formation of acid–base interaction between the acidic phenol hydroxyls of RES and dimethylamino groups of Eudragit E PO.

#### 1.2.2 Polarized Light Microscopy (PLM)

An amorphous sample does not exhibit birefringence under polarized light unlike a crystalline sample. The crystallinity of pure RES was evidenced by the birefringence of its particles by PLM. The birefringence in PM was also clear, compared to faded particles in SD, suggesting amorphization of RES in the SD (Supporting Information).

#### 1.2.3 Scanning Electron Microscopy (SEM) and Particle Size Distribution (PSD)

**Figure 5:**
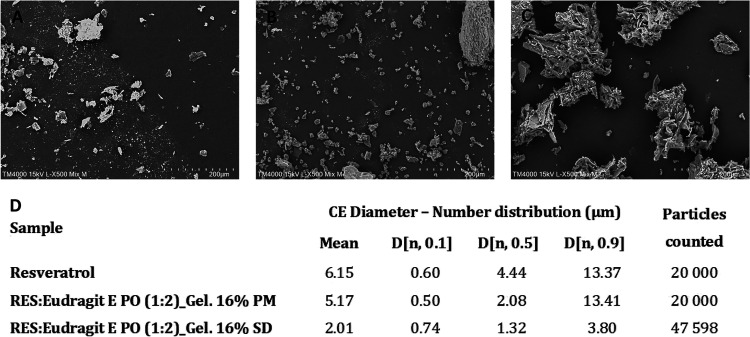
SEM of RES (A), RES:Eudragit E PO (1:2)_Gelucire 44/14 16% PM (B), and RES:Eudragit E PO (1:2)_Gelucire 44/14 16% SD (C). CE diameter calculated parameters determined by Morphologi 4 Malvern Panalytical (D).

RES particles tend to form agglomerates (Figure 5A). The SEM micrographs of PM revealed particles with smooth contours, (Figure 5B), while the SD particles are more porous with rough contours (Figure 5C). Compared to visible RES isolated particles in PM, the drug disappeared in the SD, suggesting that it could have been converted to the amorphous state dispersed in the polymer (Figure 5C).

Automated morphological imaging was carried out using a Morphologi 4 Malvern Panalytical (Worcestershire, England), to provide a detailed description of the morphological properties of the particles. The circle equivalent (CE) diameter, defined as the diameter of a circle with the same area as the 2D image of the particles was assessed, and results are presented in Figure 5D. The preparation of a third-generation SD by the lyophilization method was able to origin particles with more than a 3-fold decrease in mean particle size, D_50_, and D_90_, compared to RES alone.

#### 1.2.4 Solubility

**Figure 6:**
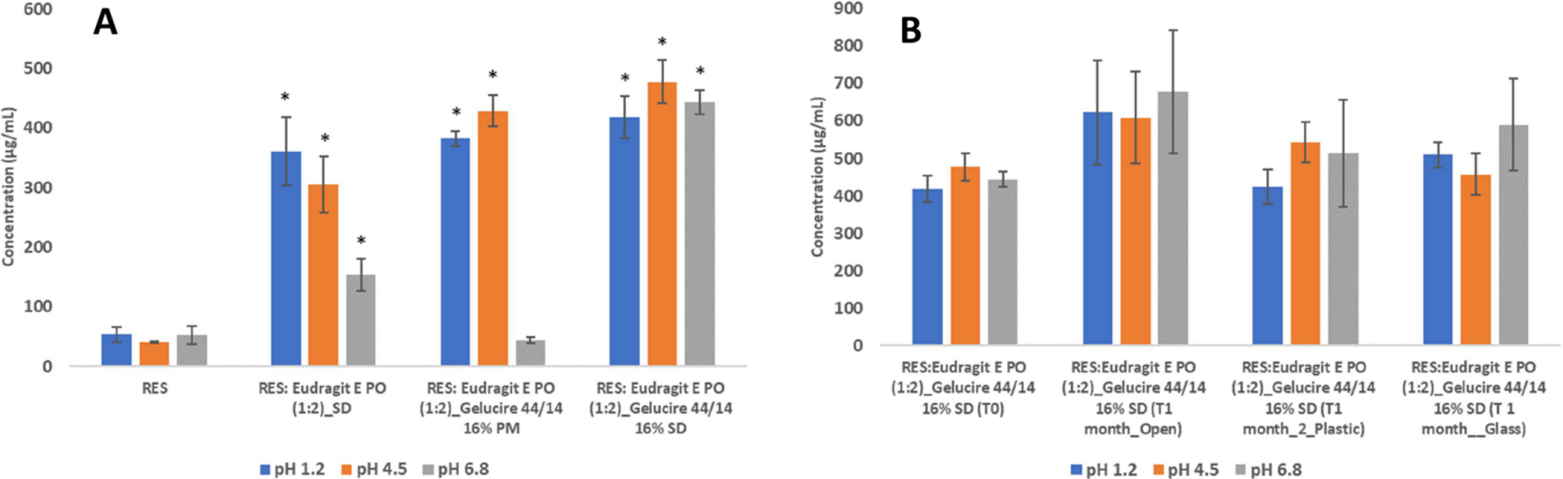
Solubility after 24 h under magnetic stirring at room temperature in pH 1.2, 4.5, and 6.8 at T0 (A), and after 1 month at 40 °C/75% RH (B) (mean ± SD, *n* = 3), *p < 0.05 comparing with pure RES.

The lyophilized formulation with Gelucire 44/14 (third-generation SD) presented greater solubility than the formulation with only Eudragit E PO (second-generation SD) in all tested buffers. Third-generation SD presented an 8-, 12-, and 8-fold increase of RES solubilized compared to pure RES at pH 1.2, 4.5, and 6.8, respectively (Figure 6A). At pH 6.8, a 10-fold increase in solubility compared to the PM was observed. Eudragit E PO being a cationic polymer precipitates above pH 5.5, explaining the lower solubility of RES obtained in the PM at pH 6.8 (Figure 6A). This is also evidence of a chemical interaction in the SD compared to the PM.

The solubility of the third-generation SD without packaging and packaged in plastic and amber glass bottles remained relatively constant in all buffer solutions after 1 month of storage at 40 °C/75% RH (Figure 6B). These results indicate that the third-generation SD appears to be stable.

#### 1.2.5 Dissolution

**Figure 7:**
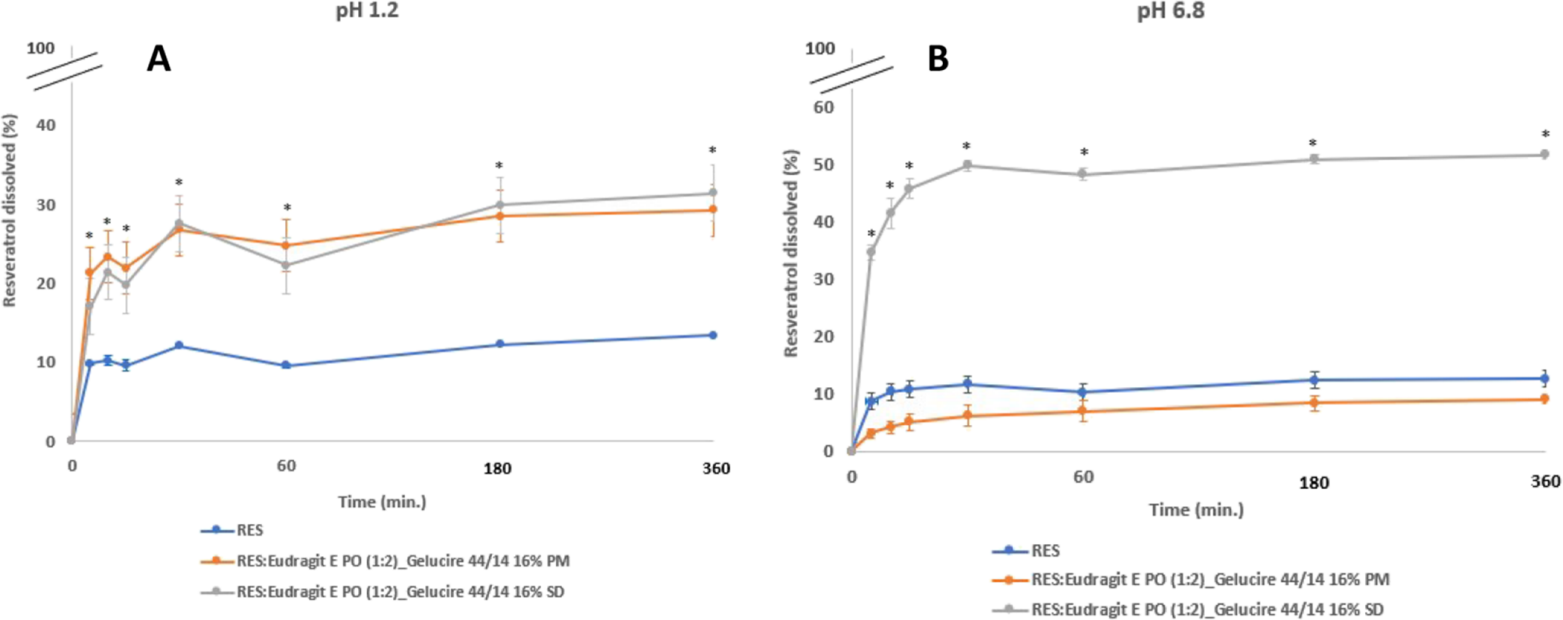
Dissolution profile of RES, PM, and SD powders, at 37 °C in pH 1.2 (A), and pH 6.8 (B). RES = 400 μg/mL, in each sample. (mean ± SD, *n* = 3), *p < 0.05 comparing PM and SD with pure RES in pH 1.2 and comparing SD with RES and PM in pH 6.8.

At pH 1.2, as expected, RES presented the lowest rate and extent of dissolution, due to its low solubility. Under these acidic conditions, as observed for solubility assessment, third-generation SD and its PM exhibited a similar dissolution rate with more than 2-fold increase in RES dissolved compared to RES alone after 30 min (Figure 7A).

Furthermore, in line with results obtained for solubility assessment at pH 6.8, the increase in RES dissolution for the third-generation SD was clear, compared to PM and RES alone at pH 6.8, with almost 5-fold increase compared to RES alone after 30 min (Figure 7B).

RES conversion from the crystalline to the amorphous state, particle size reduction, weakening of aggregation and agglomeration, solubilizing effect of the polymer, and wettability improvement are some mechanisms that can explain the dissolution and solubility improvement in the third-generation SD compared to PM and pure RES.

### 1.3 Formulation Biological Assessment

To confirm whether the developed third-generation SD allowed to obtain an enhanced RES bioavailability compared to its PM and second-generation SD, an in vitro study in cell-based models for intestinal permeability, and an in vivo pharmacokinetic study in Wistar rat model were conducted.

#### 1.3.1 In Vitro Intestinal Permeability

**Figure 8:**
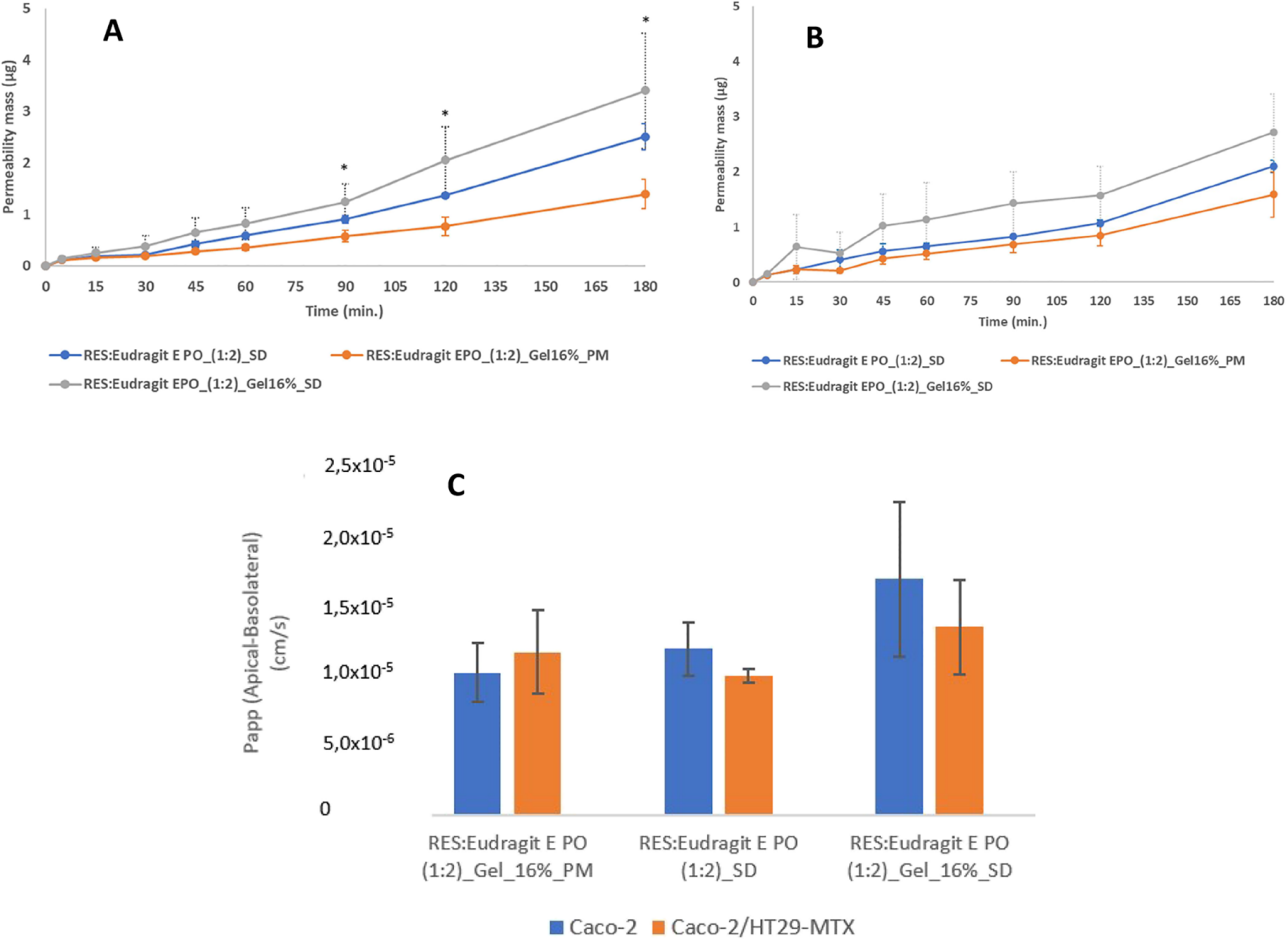
RES permeability in Caco-2 cell-based intestinal in vitro model (A), and in Caco-2/HT29-MTX cell-based intestinal in vitro model (B). Apparent permeability (*P*_app_) of RES third-generation SD, PM, and second-generation SD (C). RES theoretical concentration administered in each sample: 50 μg/mL. (mean ± SD, *n* = 3), *p < 0.05 comparing third-generation SD with PM.

In both cell models, third-generation SD presented higher permeability, compared to its PM and second-generation SD (Figure 8). Despite the presence of mucus-secreting cells (HT29-MTX), similar results were obtained for both models, suggesting no interference of mucus in the performance of third-generation SD and consequently enhanced permeability of RES.^36^ In Caco-2, at 90 min and following time points the difference between third-generation SD and its PM was statistically significant (Figure 8A). At 180 min the average RES permeated was 2.4- and 1.7-fold higher in Caco-2 and Caco-2/HT29-MTX respectively compared to its PM (Figure 8A,B). Apparent RES permeability was higher with third-generation SD in both cell models, as presented in Figure 8C.

The improved RES permeability observed in the third-generation SD, compared to its PM can be explained by the physical and chemical interaction of RES with Eudragit E PO and Gelucire 44/14 in the SD. Gelucire 44/14 primary mechanism to increase the bioavailability of orally administered low soluble drugs like RES is through improved dissolution rates in the gastrointestinal tract. Furthermore, there is increasing evidence that Gelucire 44/14 can inhibit presystemic drug metabolism and can reduce P-gp mediated efflux, which contributes to improved RES bioavailability.^26,29,30^ These two additional mechanisms can also have contributed to the higher RES permeability from the third-generation SD compared to the second-generation SD tested.

The mechanism by which excipients like Gelucire 44/14 inhibit P-gp activity is currently unknown; however, theories include altering cell membrane integrity, blocking binding sites competitively, noncompetitively, or allosterically, interfering with ATP hydrolysis and creating a futile cycle of ATP hydrolysis.^26,29,30^

#### 1.3.2 In Vivo Pharmacokinetic Assessment

**Figure 9:**
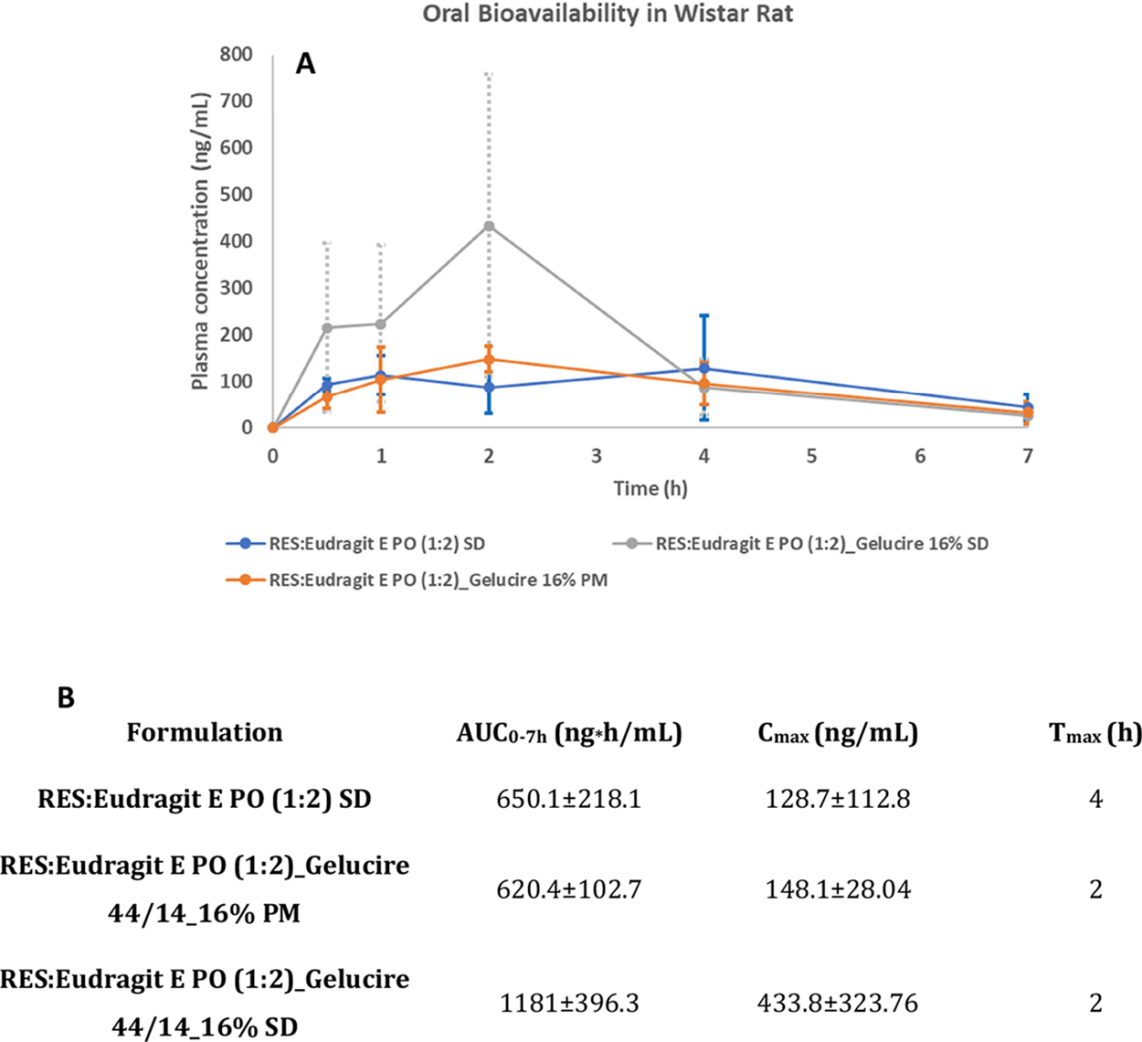
Plasma concentration–time profiles of RES third-generation SD, PM, and second-generation SD after oral administration of samples containing 200 mg/kg of RES (A). Pharmacokinetic parameters for RES third-generation SD, PM, and second-generation SD in Wistar rats (B) (mean ± SD, *n* = 5).

The third-generation SD was able to unquestionably enhance RES bioavailability, while its PM and second-generation SD presented a very similar pharmacokinetic profile (Figure 9A). In the third-generation SD formulation, higher RES concentration in plasma compared to PM and second-generation SD was observed immediately after oral administration, with a 2.3- and 3.2-fold increase, respectively, after 30 min. After 60 min, the RES permeated with third-generation SD was 2.0- and 2.2-fold higher compared to second-generation SD and PM respectively. After 4 h RES plasma concentration in the third-generation SD decreased to values similar to those in the other 2 formulations and continued to decrease to lower values after 7 h.

The third-generation SD clearly presented the highest values for *C*_max_ with a 1.9- and 1.8-fold increase compared to its PM and second-generation SD. The third-generation SD was also 2.9- and 3.4-fold higher in AUC_0–7h_, than PM and second-generation SD respectively (Figure 9B). As previously discussed with regards to in vitro intestinal permeability assessment, the higher RES bioavailability obtained with the third-generation SD over the other 2 formulations, can be explained not only by the dissolution improvement, but also by the potential interference of Gelucire 44/14, with RES metabolism, and inhibition of P-gp mediated efflux. The presence of excipients like Gelucire 44/14 in the SD allows for greater bioavailability of orally administered RES.^26,29,30^ Increased RES permeability and bioavailability by surfactants (Gelucire 44/14 and poloxamer 407) in SDs due to reduction of efflux transport and metabolism, had already been demonstrated by Vasconcelos T, Prezotti F, Araújo F, Lopes C, Loureiro A, Marques S, Sarmento B.^13^ In this study, a RES:Soluplus (1:2)_poloxamer 407_15% SD was administered to rats at a RES dose of 100 mg/kg, obtaining an AUC_0–7h_ of 279 ± 54 ng·h/mL, and a *C*_max_ of 134 ± 78 ng_/_mL. The third-generation SD developed in the current work, allowed an increase of more than 2- and 1.5-fold respectively for both parameters (corrected values considering different doses administered in the two studies), compared with the formulation developed by those authors.

P-gp is a 170-kDa transmembrane protein member of the ATP binding cassette (ABC) transporter family, which utilizes energy released by ATP hydrolysis. It is localized at the apical secretory surface of various tissues (e.g., liver, kidney, gastrointestinal tract, blood-brain barrier) where it mediates the active transmembrane transport of a variety of lipophilic substrates, which tend to be large, aromatic, and amphiphilic. P-gp can extrude/exclude a wide range of structurally diverse xenobiotics and limits the oral absorption of several drugs such as RES by transporting them back from the intestinal cells into the gut lumen.^26,29,30,37^

There is increasing evidence that some lipid excipients like Gelucire 44/14 are capable of inhibiting P-gp-mediated drug efflux back to the intestine.^13,14,26^ Several mechanisms have been proposed. Surfactants have been shown to modulate P-gp activity by changing the fluidity of the lipid membrane environment of P-gp leading to a reduction of ATPase activity.^38,39^ Other study showed that Pluronic block copolymers sensitize multidrug resistance cell lines by decreasing the affinity of P-gp for ATP, decreasing the ATPase activity in combination with depletion of intracellular ATP.^40^ Lipid excipients like Gelucire 44/14 actively affect P-gp efflux mechanism not only by altering the membrane fluidity or ATPase activity but also by downregulation of P-gp expression.

Gelucire 44/14, presystemic drug metabolism inhibition, and interference in the P-gp efflux mechanism also contributed to the higher *C*_max_ and AUC in plasma observed with the third-generation SD. Protecting RES from rapid metabolization in the gastrointestinal tract and liver is also a general mechanism that can increase bioavailability. Given that CYP, UGT, and SULT are the key enzymes that conjugate RES, any intervention that decreases their rate of reaction to it should increase the concentration of the parent compound.^12^

Strategies to increase and extend in time RES permeability by SD formulation using the same excipients, would encompass the use of a higher quantity of Gelucire 44/14 in the SD and consequently higher inhibition of efflux mechanisms due to more available surfactant and increased RES metabolism interference. Alternatively, other surfactants with known efflux pump inhibition such as Tweens or Pluronics, polysaccharides, polyethylene glycols and derivates, amphiphilic block copolymers, dendrimers, and thiolated polymers, should be investigated to assess their performance.^41^

## 2 Conclusions

In the presented study, a resveratrol (RES) third-generation solid dispersion (SD) formulation was developed and produced by lyophilization, containing Eudragit E PO as the hydrophilic carrier and Gelucire 44/14 as a surfactant to maximize the solubility and dissolution of RES and consequently enhance its oral bioavailability. RES:Eudragit E PO was used in a 1:2 ratio. Gelucire 44/14 was present at 16% (w/w) of RES.

RES conversion from the crystalline to the amorphous state, particle size reduction, increased porosity, weakening of aggregation and agglomeration, solubilizing effect of the polymer, and wettability improvement are some mechanisms that explain the solubility and dissolution improvement observed with the third-generation SD compared to physical mixture (PM) and pure RES. Enhanced oral bioavailability was obtained not only by solubility and dissolution improvement, but also by the interference of Gelucire 44/14 with RES metabolism, and inhibition of P-gp mediated efflux. The presence of excipients like Gelucire 44/14 in the SD allows for greater bioavailability of orally administered RES, making it easier to obtain some of the physiological benefits demonstrated by this molecule.

## 3 Materials and Methods

### 3.1 Materials

Resveratrol was provided by Bial–Portela & Ca̲ S.A., and was manufactured by Abatra Technology, China. Acetic acid R, Acetonitrile (ACN), Dimethyl sulfoxide, Glacial acetic acid R, Hydrochloric acid R at 37%, Polyethylene glycol **(**PEG 10000), Potassium dihydrogen phosphate monohydrate R, Sodium acetate trihydrate R, Sodium dodecyl sulfate (SDS), Sodium hydroxide R and *tert*-butanol (TBA) were acquired from Merck, Germany. Cationic methacrylate copolymers (Eudragit E100) and (Eudragit E PO) were obtained from Evonik, Gertmany. Lactose 200 was purchased from Meggle, Germany. Cetrimide was purchased from Glentham Life Sciences, Germany. Compitrol 888 ATO, Gelucire 44/14, and Labrasol were obtained from Gattefossé, France. Copovidone (Plasdone S-630) was purchased from Ashland, USA. Kolliphor RH 40, Polaxamer 407, polyvinyl caprolactam-polyvinyl acetate-polyethylene glycol graft copolymer (Soluplus), and polyvinylpyrrolidone (Povidone K30) were purchased from Basf, Germany. Docusate sodium was purchased from Solvay, China. Dulbecco’s Modified Eagle medium (DMEM), Hank’s balanced salt solution (HBBS), Heat inactivated fetal bovine serum (FBS), l-glutamine, Nonessential amino acids (NEAA), Penicillin (10000 IU/mL), Streptomycin (10 mg/mL) and Trypsine-EDTA were obtained from HyClone; USA. Hydroxypropyl cellulose Low Viscosity (HPC SL; MW: 806.9) was obtained from Nippon Soda, Japan. Hydroxypropyl methyl cellulose (HPMC; MW: 1261.4) was purchased from Colorcon, UK. Hypromellose Acetate Succinate (HPMC AS-MG) was purchased from Ashland, USA. Mannitol was purchased from Cargil SRL, Italy. Tween 80 was purchased from Croda, UK. Water R was obtained from a Milli-Q water system. All other reagents used were of analytical grade.

### 3.2 Preparation of Solid Dispersions through Lyophilization

Eudragit E PO was solubilized in acetate buffer pH 4.5. RES was solubilized in TBA. Both solutions were combined and Gelucire 44/14 was added under agitation with an overhead stirrer RW 20 IKA (Wilmington, USA) until complete dissolution. A RES:Eudragit E PO (1:2)_Gelucire 44/14 16% in TBA/Acetate buffer pH 4.5 (75:25) solution was obtained to be freeze-dried. Batch lyophilization in vials and bulk lyophilization in the aluminum tray were performed in an SP Scientific – Advantage Pro EL lyophilizer (Virtis, USA).

A physical mixture (PM) of the third-generation SD, and a second-generation SD composed of RES:Eudragit E PO (1:2) without the surfactant were also produced for comparison purposes, and to assess whether Gelucire 44/14 has any influence on in vitro permeability and in vivo pharmacokinetics of RES.

### 3.3 Solid-State Characterization

Differential scanning calorimetry (DSC), scanning electron microscopy (SEM), Fourier-transform infrared spectroscopy (FTIR), polarized light microscopy (PLM), X-ray powder diffraction (XRPD), and particle size distribution (Morphologi 4 Malvern Panalytical) were selected for solid-state characterization (Supporting Information).

### 3.4 Solubility

An excess amount of RES, PM, or SD was added to a 4 mL vial; 2.5 mL of buffer was added to each vial and strongly agitated in a vortex mixer for 30 s to facilitate appropriate mixing. Samples were then maintained under magnetic stirring at room temperature (15–25 °C) for 24 h. Aqueous solvent buffers at pH 1.2, 4.5, and 6.8 were used. Each sample was prepared in triplicate. After stirring, suspensions were filtered through a 0.45 μm filter and analyzed by HPLC.

### 3.5 Dissolution

Dissolution study of RES, PM, and SD was performed using a Teledyne Hanson Vision Elite 8 dissolution tester apparatus 2 (Teledyne Hanson, Chatsworth, USA), with a paddle rotation speed of 100 rpm at 37 ± 0.5 °C in 500 mL of pH 1.2 and pH 6.8 buffer solutions. Samples equivalent to 200 mg of RES were added to the equipment vessels; [RES = 400 μg/mL]. Five mL samples were withdrawn at 5, 10, 15, 30, 60, 180, and 360 min. Samples were centrifuged at 3500 rpm for 5 min, and the supernatant was analyzed by HPLC. Each sample was prepared in triplicate.

### 3.6 In Vitro Intestinal Permeability

An in vitro study to assess intestinal permeability of RES in PM, second-generation SD, and third-generation SD was conducted in Caco-2 monoculture and Caco-2/HT29-MTX dual coculture cell models. For the permeability experiments, 1 × 10^5^ cells/cm^2^ of Caco-2 and Caco-2/HT29-MTX (9:1) were seeded in 12-Transwell cell culture inserts and were allowed to grow and differentiate for 21 days at 37 °C in a carbogen (95% O_2_, 5% CO_2_) atmosphere, with the culture medium replacement every other day.^42^ After that time, the medium was carefully removed from the apical and basolateral compartments, and the insets were gently washed twice with phosphate-buffered saline (PBS) (pH 7.4, 37 °C). Then, 0.5 and 1.5 mL of HBSS were added to the apical and basolateral parts of the Transwell, respectively, and allowed to equilibrate for 30 min inside the incubator. Afterward, the media from the apical compartment was removed and 0.5 mL of each sample previously dissolved in DMSO with a RES theoretical concentration of 50 μg/mL in HBSS was added. The test samples were placed directly in the apical compartment without removing the media. Triplicate samples and an insert without the addition of a sample were used as a control in both models. Plates were placed inside an orbital shaking incubator (KS 4000 IC, IKA, Staufen, Germany) at 100 rpm and 37 °C. Aliquots (200 μL) were withdrawn from the basolateral chamber at predetermined times (5, 15, 30, 45, 60, 90, 120, and 180 min.) and immediately replaced with HBSS. At the end, an aliquot from the apical compartment was collected.^42^ Before, during, and at the end of the permeability experiments, the transepithelial electrical resistance (TEER) was measured using an EVOM^2^ epithelial voltammeter (WPI) with chopstick electrodes (World Precision Instruments, Sarasota, FL, USA) to monitor the formation, confluence, and integrity of the cell monolayers. The concentration of RES in the samples was determined by HPLC-UV analysis.

### 3.7 In Vivo Pharmacokinetics

An in vivo study to assess RES pharmacokinetics in PM, second-generation SD, and third-generation SD was conducted using male Wistar rats, weighing approximately 250 g, purchased from Charles River Laboratories (France). The animal study protocol complied with the guidelines from Directive 2010/63/EU of the European Parliament on the protection of animals used for scientific purposes and the Portuguese law on animal welfare (*Decreto-Lei 113/2013*). Three groups of rats (one per formulation) of 5 animals each were tested. Rats were randomly separated into 5 animals per cage and placed on a wood litter, with free access to a pellet chow diet (2014 Envigo) and tap water. The animal cages were maintained in a 12-h light/dark cycle (07:00 to 19:00 h) in a controlled ambient temperature of 22 ± 2 °C and relative humidity of 50 ± 20%. The day before food was removed. On the day of administration, rats were weighed, and each formulation was orally administered via gastric gavage at a dose of 200 mg/kg of RES via dispersion state in 6 mL of hydroxypropyl methylcellulose 0.5% (w/v) in water before dosing. Approximately 150 μL of blood samples were collected by lateral tail vein at predetermined time points (predose, 0.5, 1, 2, 4 h), with the exception of the last time point (7 h), in which samples were collected by cardiac puncture, after an overdose of pentobarbital. The last time point (7 h) was selected instead of the usual 8 h, due to logistical laboratory constraints. Plasma was isolated through centrifugation at 1500 rpm for 15 min (4 °C) and samples were stored at −80 °C. Samples were assayed for RES by liquid chromatography with tandem mass spectrometry (LC-MS/MS). The *C*_max_, *T*_max,_ and AUC_0–7h_ were calculated for each group using GraphPad Prism (GraphPad Software Inc., CA, USA).

### 3.8 Statistical Analysis

For solubility, dissolution, and in vitro permeability determinations, triplicates of formulations were analyzed with Microsoft Excel 2016. Student’s *t*-test was used between pairs of experiments using a two-tailed distribution with two-sample equal variance. Pairs were considered statistically different with *p* values below 0.05. For pharmacokinetics, Student’s *t*-test for pairs of samples and one-way analysis of variance for all tests (ANOVA) with unpaired and Bonferroni posthoc test (GraphPadPrism, GraphPad Software Inc., CA, USA) were used to analyze the data, respectively. The level of significance was set at probabilities of *p* < 0.05.

## Supporting Information Available

Methods description; DSC, XRPD; FTIR; PLM; SEM; PSD; in vitro intestinal permeability; resveratrol solid dispersion development; selection and optimization of hydrophilic carrier content; selection and optimization of surfactant content; Solid Dispersions Characterization; and PLM (PDF)
