# Supplementary material for: Third-Generation Solid Dispersion Through Lyophilization Enhanced Oral Bioavailability of Resveratrol
Source: ACS Pharmacol Transl Sci. 2024 Feb 14;7(3):888–98. doi: 10.1021/acsptsci.4c00029 (PMC10928883; doi:10.1021/acsptsci.4c00029)
Supplement: Supplementary file 1 — pt4c00029_si_001.pdf [file pt4c00029_si_001.pdf]

Third Generation Solid Dispersion through Lyophilization Enhanced Oral Bioavailability of Resveratrol

Hugo Almeida[1,2,3,4], Bárbara Ferreira [1,3,4], Carlos Fernandes-Lopes[2], Francisca Araújo[2], Maria João Bonifácio[2], Teófilo Vasconcelos[2], Bruno Sarmento[*3,4,5]

[1] ICBAS – Instituto de Ciências Biomédicas Abel Salazar, Universidade do Porto, Rua Jorge de Viterbo Ferreira, 228, 4050-313 Porto, Portugal

[2] BIAL – Portela & Cª, S.A., Avenida da Siderurgia Nacional, 4745-457 Trofa, Portugal

[3] INEB – Instituto Nacional de Engenharia Biomédica, Universidade do Porto, Rua Alfredo Allen, 208, 4200-135, Porto, Portugal

[4] i3S – Instituto de Investigação e Inovação em Saúde, Universidade do Porto, Rua Alfredo Allen, 208, 4200-135, Porto, Portugal

[5] IUCS - CESPU, Rua Central de Gandra 1317, 4585-116 Gandra, Portugal

1. Methods

1.1. Differential scanning calorimetry (DSC)

Thermal analysis was performed in a TA Instruments Q200 calorimeter (TA Instruments, USA). Approximately 2-10 mg samples (RES, PM and SDs), were packed into a 100 μL aluminum pan with a lid. Each sample was heated from 25ºC to 300ºC at a heating rate of 5ºC/min. under nitrogen atmosphere (50 mL/min.).

1.2. X-ray powder diffraction (XRPD)

The molecular conversion of RES from crystalline to amorphous state was assessed by XRPD. RES, PM and SD samples were measured using a D/tex Ultra2 X-ray powder diffractometer (Rigaku-Miniflex 600; Tokyo, Japan). A Cu Kα radiation was used at 40 KV and 15 mA. The samples were scanned in the reflection mode from 3º to 40º at a scan speed of 5.00º/min. and a scanning step size of 0.02º.

1.3. Fourier transform infrared spectroscopy (FTIR)

The presence of molecular interactions between RES and hydrophilic carrier was determined with a Bruker Tensor 27 Fourier transform infrared spectrometer (Karlsruhe, Germany). The attenuated total reflection (ATR) technique was used to obtain the spectrum from each sample. Before each measurement, the ATR crystal was carefully cleaned with isopropanol. During the measurement, the sample was in contact with the universal diamond ATR top-plate. For each sample, the spectrum representing an average of 16 scans was recorded in the range of 4000-400 cm^{-1} with a 4 cm^{-1} resolution. Data obtained was analysed with Spectragryph 1.2 software (Informer Technologies, Inc.; Dominica).

1.4. Polarized light microscopy (PLM)

The shape, size and crystallinity of the particles was observed using polarized light microscopy analysis. A Motic BA410E polarizing photomicroscope (Motic®, China) was used for analysing the samples. Briefly, the powdered sample was spread out evenly on a glass slide to avoid clumping. The excess powder was dusted off. The slide was then placed onto the microscope stage and observed under a 400X magnification. Presence of birefringence, a property observed in crystalline substances was considered as an indication of the presence of crystallinity. Images were captured using Moticam 3+ digital camera (Motic®, China) under light using dark background conditions. Captured images were analysed using Motic® Image Plus 3.0 software (Motic®, China).

1.5. Scanning electron microscopy (SEM)

The surface morphology of samples was examined using a Hitachi® TM400 Plus scanning electron microscope (Tokyo, Japan). A small amount of sample was sprinkled onto a double sided electrically conductive adhesive tape mounted on an aluminium stub, coated with platinum, and then examined under vacuum using an ion sputter to provide electrical conductivity. Images were acquired at a beam acceleration voltage of 15 KV under a 500X magnification.

1.6. Particle size distribution (PSD)

Automated morphological imaging was carried out using a Morphologi 4 Malvern® Panalytical (Worcestershire, England). This was used to investigate particles size. Samples were prepared by dry powder dispersion using compressed air. A total of 20 000 particles were analysed for RES and PM samples and 47 598 particles for the third-generation SD.

1.7. *In vitro* intestinal permeability

The Caco-2 and HT29-MTX cell lines were obtained from the American Type Culture Collection (ATCC, Manassas, VA, USA). Both Caco-2 cell line and co-culture Caco-2/HT29-MTX (9:1), were cultured in Dulbecco's Modified Eagle Medium (DMEM) containing 10% fetal bovine serum, 1% (v/v) non-essential amino acids, 2 mM L-glutamine, 100 µg/mL streptomycin and 100 U/mL penicillin. Both models were sub-cultured once a week using 0.25% Trypsin-EDTA (0,25%/0,05%), to detach the cells from the flasks and seeded at a density of 0.5 x 106 cells per 75 cm^2 flasks. The culture medium was replaced every other day. Cells were maintained at 37 °C, 5% CO_2 and 95% relative humidity.

RES apparent permeability (Papp) was calculated using the formula below:

$$P_{app} = \frac{dQ/dt}{A \cdot C_0}$$

Equation 1 – Apparent permeability

Where Papp is the apparent permeability (cm/s); dQ/dt, (µM/s): is the rate of permeation across the monolayer obtained from the angular coefficient of the curve of the amount of drug transported versus time; V, (cm^3): is the acceptor chamber volume, which in this case corresponds to 1.5 cm^3 (basolateral chamber); A, (cm^2): is the insert membrane growth area (equal to 1.1 cm^2 for a 12 well plate); and C_0 (µM) is the initial concentration of RES in the apical compartment [1].

2. Results

2.1. Resveratrol Solid Dispersion Development

2.1.1. Selection and optimization of hydrophilic carrier content

Aiming to develop a RES drug delivery system with improved solubility and consequently enhanced oral bioavailability, by the solvent evaporation method (Lyophilization/Freeze-drying), several hydrophilic polymers were initially screened and characterized with the purpose to disperse the RES at a molecular level and consequently induce its solubility improvement. Non-ionic polymers: Polyethylene glycol **(**PEG 10000); Polyvinylpyrrolidone (Povidone K30)**;** Copovidone (Plasdone S-630)**;** Polyvinyl caprolactam-polyvinyl acetate-polyethylene glycol graft co-polymer (Soluplus)**;** Hydroxypropyl methyl cellulose (HPMC;

MW: 1261.4) and Hydroxypropyl cellulose Low Viscosity (HPC SL; MW: 806.9). Cationic polymer: Cationic methacrylate copolymer (Eudragit E100). Anionic polymer: Hypromellose Acetate Succinate (HPMC AS-MG).

2.1.1.1. Hydrophilic carrier selection

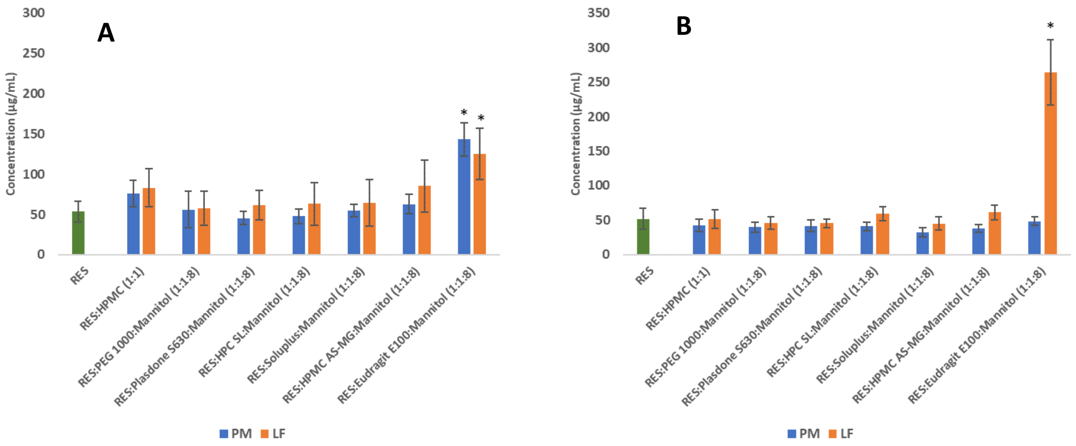

Figure S1 – Solubility after 24 hours under magnetic stirring at room temperature in pH 1.2 – A and pH 6.8 – B, (high throughput polymer screening). (mean±SD, *n*=3), $^{*}p < 0.05$ comparing with pure RES.

2.1.2. Selection and optimization of surfactant content

2.1.2.1. Surfactant selection

Compitrol 888 ATO, Docusate sodium, Gelucire 44/14, Polaxamer 407, Tween 80, Labrasol, Cetrimide, Sodium dodecyl sulfate (SDS) and Kolliphor RH 40 were the surfactants tested at 4% (w/w) to RES. Solubility in pH 1.2 aqueous media for LFs was assessed at T0 and after 1 month at 40ºC/75%RH (Figure 2).

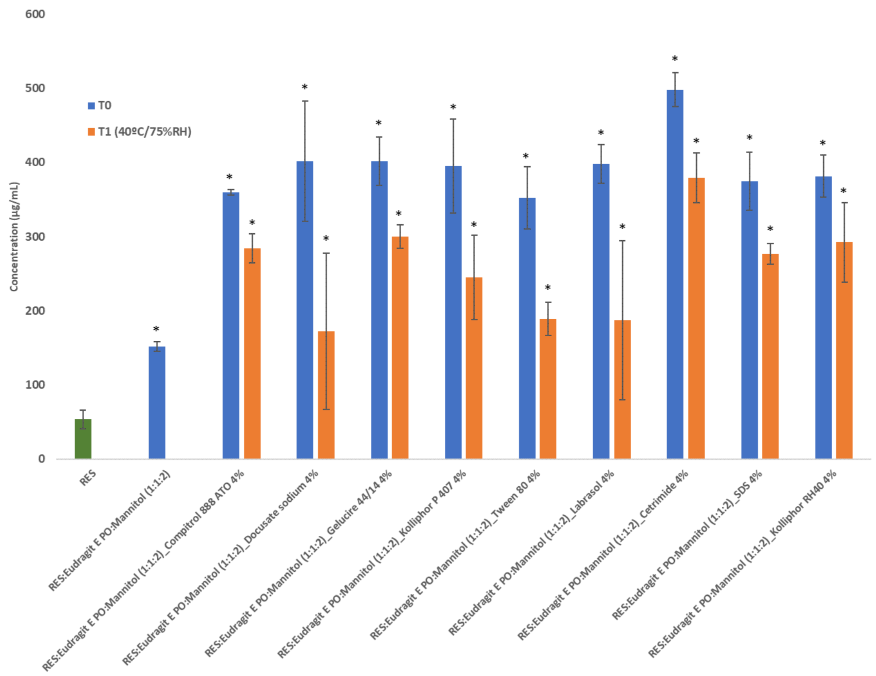

Figure S2 – Solubility after 24 hours under magnetic stirring at room temperature in pH 1.2 at T0 and T1 month at 40ºC/75% RH, (high throughput surfactant screening). (mean±SD, *n*=3), $^{*}p$ < 0.05 comparing with pure RES.

2.2. Solid Dispersions Characterization

2.2.1. Polarized light microscopy (PLM)

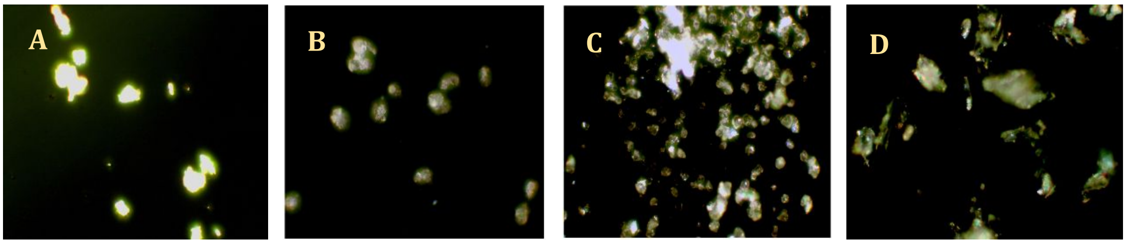

Figure S3 - Polarized light microscopy of RES – A, Eudragit E PO – B, RES:Eudragit E PO (1:2)_Gelucire 44/14 PM – C, and RES:Eudragit E PO (1:2)_Gelucire 44/14 SD – D.

3. References

1. Antunes F, Andrade F, Araújo F, Ferreira D, Sarmento B. Establishment of a triple co-culture in vitro cell models to study intestinal absorption of peptide drugs. Eur J Pharm Biopharm. 2013;83(3):427-35. 10.1016/j.ejpb.2012.10.003.
